# Digital Video Broadcast-Terrestrial (DVB-T) Single Frequency Networks Positioning in Dynamic Scenarios

**DOI:** 10.3390/s130810191

**Published:** 2013-08-09

**Authors:** Jie Huang, Letizia Lo Presti, Roberto Garello

**Affiliations:** Politecnico di Torino, Corso Duca degli Abruzzi 24, Turin 10129, Italy

**Keywords:** DVB-T, signal-of-opportunity, GNSS, positioning

## Abstract

Since Global Navigation Satellite Systems (GNSS) show degraded performance in dense urban and indoor areas, a positioning sensor based on Digital Video Broadcast-Terrestrial (DVB-T) systems is presented in this paper. DVB-T signals can be considered as signals-of-opportunity for positioning, due to their good properties. One of the challenges to overcome is to distinguish the signals from different emitters. Here, we suppose that the user can first compute his position by GNSS during an initialization phase, which is used for solving all the ambiguities concerning DVB-T emitters. Starting from there, DVB-T signals can be used for aiding positioning when the user enters a GNSS-blocked area, up to a limit case, where all the GNSS satellites are not in view and only DVB-T signals are used for positioning. We tested this method by simulation, by adopting the Hata model for the emitter attenuations and the Rayleigh model for multipath. The obtained results show good performance if the receiver correctly associates the signal to the user's motion.

## 1. Introduction

Global Navigation Satellite Systems (GNSSs) have been widely used in many applications for positioning, navigation and timing. However, in urban areas and indoor environments, where position information is required for many applications, GNSSs show degraded performance in terms of precision and availability, because of the signal loss or attenuation and multipaths due to obstacles. Fortunately, in these areas, many local networks are deployed, such as 2G, 3G, WiFi, LTE (Long Term Evolution) and DVB-T (Digital Video Broadcast-Terrestrial). They were originally designed for other purposes, but they can be used for positioning, thanks to their properties, such as a high signal-to-noise ratio (SNR).

In this paper, we consider DVB-T systems. DVB-T is the European digital TV standard. It adopts the Orthogonal Frequency Division Multiplexing (OFDM) technique. Therefore the whole bandwidth is divided into many subcarriers, in which the pilot subcarriers are included. We used these pilot subcarriers to estimate the ranges between the receiver and different emitters with a mechanism similar to the one used by GNSS receivers. Since the SNR requirement for ranging is much lower than the one required by TV service, the receiver is able to see several emitters in one point. If three or more signals are successfully processed, the receiver can provide a DVB-T only the positioning; otherwise, it can be used to assist GNSS.

According to the document [1], two different network types are introduced, Multi-Frequency Network (MFN) and Single Frequency Network (SFN). In MFN, different emitters transmit the same signal on different frequencies, and they are not exactly synchronized; while in SFN, all the emitters simultaneously transmit the same signal in the same frequency. The synchronization is provided by some professional GPS timing receivers, and this represents a key point when DVB-T signals are used for positioning purposes. Notice that the transmission of the emitter identifier (ID) is optional: this is not an issue for the provision of the DVB-T service, but it is a problem to be solved when the signals are used for positioning.

It is interesting to note that some companies transmit the emitter ID within their bit streams, which are then completely equal apart from this small difference (this is done, for example, by Rai in Italy). This causes a (very limited) penalty, but is very useful for network management and control. It is clear that this extra-information can be very useful also for positioning purposes, since it highly simplifies the association of each echo to the corresponding emitter. Anyway, since the ID emitter transmission is optional in the DVB-T standard (which is much more used and available than DVB-Handheld (DVB-H)), we have not considered it in the study, and we have proposed another way to solve the ambiguity. (Clearly, some actions could be adopted in the future to convince the TV companies to transmit it, to further exploit DVB-T for positioning.)

The scenario analyzed in this paper considers a position device (PD) consisting of a hybrid GNSS/DVB receiver, where GNSS is the primary positioning system, and DVB-T is used as a back-up when the number of GNSS satellites is not sufficient for computing the receiver position. In the first phase (called the initialization phase in the rest of the paper), we suppose that the PD is able to compute its position by using GNSS only. In this phase, the PD keeps sensing the DVB-T spectrum in order to identify all the DVB-T emitters and computes and tracks the ranges between each emitter and the PD itself. Since the emitter positions are known, the ranges can be used to correctly associate each received signal to the corresponding emitter. Notice that a problem could arise when two or more ranges assume the same value; however, the probability that such an event continuously persists as long as the PD is in open sky is very low and can be neglected. When the PD enters a GNSS-hostile zone (indoor environment, urban or natural canyon or similar) and the number of visible satellites becomes lower than four, the PD can use the DVB-T ranges to integrate them, up to a limit solution, where no satellites are visible and the position is computed by using DVB-T data only. This last case obviously represents an extreme situation, which may become very fragile, in the presence of path ambiguities encountered when two ranges become equal. The problem can be quite completely eliminated if some redundancy is available or when auxiliary systems are available (*i.e.*, map matching). In Section 4, two methods are presented to solve the path ambiguity problem, with the assumption that one GNSS satellite is visible.

In the literature, the problem of using DVB signals for pseudo-range calculation is addressed in some papers. In [2], a pseudo-range measurement method in a single channel based on the DVB-T signal is presented. The method uses the classical functions of an OFDM receiver to demodulate the OFDM signal and estimate the Carrier Frequency Offset (CFO) and the Sampling Clock Offset (SCO). The method has been assessed in [3,4] through experimental results using the French digital TV terrestrial network based on the DVB-T standard. In [5], a pseudo-range measurement method based on demodulated data is described. However, no positioning algorithm is presented, because of the fact that all the emitters in an SFN simultaneously transmit the same signal on the same frequency, and the problem of distinguishing the signals from different emitters is not addressed in the paper. A method to distinguish the emitters is proposed in [6,7], based on the idea of introducing some artificial delays on the system side of a DVB-SH (satellite services to handhelds) system. Since DVB-H and DVB-T have a similar structure, in principle, the method could be also studied for DVB-T, but since this option is not available in the current DVB-T standard, the method has been ignored in our study. In our previous works [8–10], a method to distinguish different signals in a dynamic scenario has been studied by assuming that the PD is a hybrid DVB/GNSS receiver working in DVB-mode after an initialization phase provided by GNSS. In this phase, the initial position and velocity are available and represent the key point to allow the trilateration operations based on DVB-T signals. In [8], DVB-T is used as an assistant of GNSS; no DVB-T-only solution is provided. In [9], only an ideal scenario with no signal attenuation and multipath has been considered. In [10], only a simple channel model is used, and no path ambiguity solutions are considered. In this paper, these aspects are taken into account, by adopting the Hata model [11] for the attenuation and a Rayleigh model for the multipath. Two different path ambiguity solutions are also explained in this paper. Moreover, the impact of unsynchronization of different emitters is also investigated. The performance in terms of position errors in different SNR is analyzed. Some other minor, but important, points are also investigated in this paper, such as Doppler effects and a windowing technique to mitigate the side lobes.

This paper is organized as follows. Section 2 presents a description of SFN DVB-T. Section 3 describes the pseudo-range estimation method. Section 4 presents two methods to solve the path ambiguity problem. Section 5 presents the simulation results. The paper ends with the conclusions and a future work description.

## 2. SFN DVB-T Description

According to the DVB-T document [1], DVB-T systems adopt the OFDM technique. Four different types of subcarriers are used, among which the pilot subcarriers exist. They are used for channel estimation in DVB-T applications, but they can be also used for positioning by correlating the incoming signal with a local-generated replica, with a method similar to that used in GNSS receivers. In order to avoid inter-symbol interference, the Cyclic Prex (CP) is introduced in the OFDM system. This property is very helpful for our positioning purpose. In the coming part, we will give a brief introduction of the SFN DVB-T system, highlighting the points that impact more on the capabilities of these signals to behave as Signals of Opportunities (SoOs) for positioning.

### 2.1. OFDM

OFDM is a digital multi-carrier modulation method. It divides the bandwidth into a large number of closely-spaced sub-bands. On each sub-band, a Quadrature Amplitude Modulation (QAM) or Quadrature Phase-Shift Keying (QPSK) is used, and all the sub-band signals are summed together. The symbol rate is the same for each subcarrier and is equal to the sub-band bandwidth. This way, the subcarriers are orthogonal, and the spectra can overlap without causing Inter-Carrier Interference (ICI) when the receiver is well synchronized. To avoid Inter-Symbol Interference (ISI) in multipath fading channels, a guard interval is inserted prior to the OFDM symbol. This interval is used to transmit an exact replica at the end of the OFDM symbol, referred to as CP. This gives the OFDM system an excellent multipath resistance: the receiver can easily avoid the ISI if the multipath time-spreading is shorter than the guard interval.

**Figure 1.**
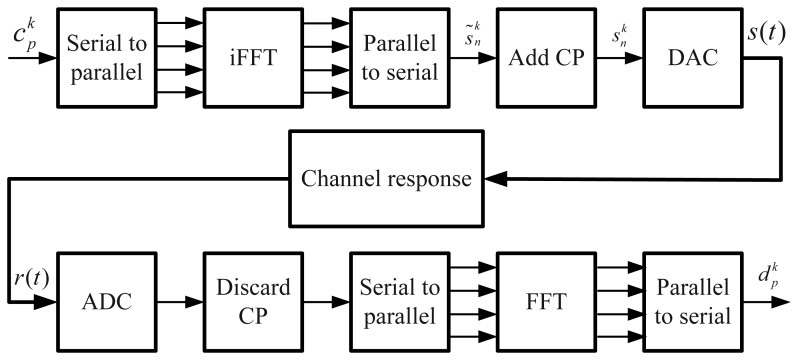
Orthogonal Frequency Division Multiplexing (OFDM) transmission system.

The Fast Fourier Transform (FFT) algorithm can be used to implement the OFDM modulation, and the good efficiency of this algorithm allows a large number of subcarriers in operation. In Figure 1, the block diagram of an OFDM transmission system is presented. The output of the parallel to the serial block can be expressed as:

(1) $$\begin{array}{c} {\tilde{s}}_{n}^{k}=\text{IFFT}(c_{p}^{k})[n]=\frac{1}{N_{\mathrm{FFT}}}\overset{N_{\mathrm{FFT}}-1}{\underset{p=0}{\text{∑}}}c_{p}^{k}\exp(j2\pi \frac{\mathrm{pn}}{N_{\mathrm{FFT}}})\\ \text{with}\;0\leq n\leq N_{\mathrm{FFT}}-1 \end{array}$$

where:

- *k* is the OFDM symbol index,
- *n* is the sample index,
- *p* is the subcarrier index,
- *N_FFT_* is the FFT size,
- ${\tilde{s}}_{n}^{k}$ is the *n*-th sample of the *k*-th OFDM symbol and
- $c_{p}^{k}$ is the data (constellation complex value) on the *p*-th subcarrier of the *k*-th OFDM symbol.

Normally, in OFDM modulation, the number of subcarriers, *N_FFT_*, is chosen as a power of two in order to efficiently use the FFT algorithm. Before the samples are transmitted, the CP is inserted at the beginning of each OFDM symbol. Therefore, the emitted signal with the CP is:

(2) $$\begin{array}{c} s_{n}^{k}={\tilde{s}}_{\mathrm{mod}(n+N_{\mathrm{FFT}}-N_{\mathrm{CP}},N_{\mathrm{FFT}})}^{k}\\ \text{with}\;0\leq n\leq N_{\mathrm{FFT}}+N_{\mathrm{CP}}-1 \end{array}$$

where:

- $s_{n}^{k}$ is the *n*-th emitted sample of the *k*-th OFDM symbol and
- *N_CP_* is the length of the cyclic prefix guard interval.

On the receiver side, the CP has to be removed. To do this, a coarsely-timed synchronization is needed to find the starting instant of the OFDM symbol. The synchronization can be achieved, for example, by using the Van de Beek algorithm [12]. This algorithm is based on the fact that the guard interval is a replica of the end of the OFDM symbol. The correlation of two groups of *N_cp_* samples spaced by *N_FFT_* is calculated, and the absolute maximum of the correlation is used to estimate the beginning of the OFDM symbol. The synchronization can be achieved more precisely by adopting a delay estimation method. If the starting point of the FFT window is slightly wrong, but still within the CP, the receiver still has all wanted samples. These samples are circularly shifted, comparing the samples originally emitted. This cyclic permutation only affects the phase of the received symbol output by the FFT. This additional phase rotation is typically compensated for by the frequency equalizer, since it is summed to the phase rotation introduced by the channel.

### 2.2. DVB-T System

DVB-T is a digital broadcasting standard created by the European Telecommunications Standards Institute. For mobile services, another available standard is DVB-H (Handheld), derived from DVB-T. In this paper, only the DVB-T is considered, but the proposed positioning technique can be also used for the DVB-H, which is more suitable for mobile users in dynamic scenarios.

**Figure 2.**
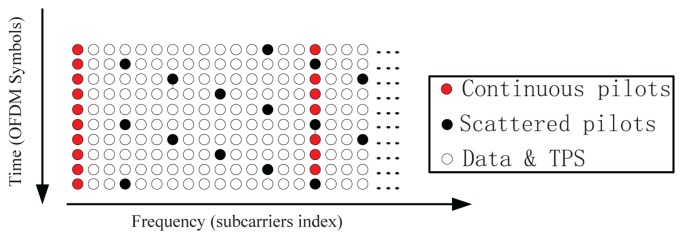
Digital Video Broadcast-Terrestrial (DVB-T) pilot organization.

The DVB-T standard family has adopted the OFDM modulation to provide high data rates along with robustness against multipath. The transmitted signal contains four types of subcarriers, as described hereafter:

- **Null** subcarriers, which are placed on the edge of the signal spectra and are not used. They serve as frequency guard bands to avoid the out-of-band emission of the OFDM signal.
- **Data** subcarriers, which are the payload of the OFDM symbol. Each subcarrier carries some data bits, depending on the modulation scheme. The data subcarriers in one OFDM frame of a DVB-T are modulated using either QPSK or 16-QAM or 64-QAM.
- **Transmission Parameter Signaling** (TPS) subcarriers, which are used for transmitting signaling parameters related to the transmission scheme. These subcarriers are Binary Phase-Shift Keying (BPSK) -modulated at the normal power level. The TPS subcarriers may contain the cell-ID information, which is the only difference between signals transmitted from different emitters in SFN. This information can be used to associate the signals to the corresponding emitters. Unfortunately, this cell-ID information is optional; the transmitters can discard this identifier by setting them to zero.
- **Pilot** subcarriers, which are used for channel estimation and equalization. They can be divided into two groups: the continuous pilots and the scattered pilots. The continuous pilots are always placed on the same subcarriers in all OFDM symbols. On the contrary, the scattered pilots are placed on different subcarriers in successive OFDM symbols. They are inserted every 12 subcarriers, and the first pilot is placed on one of four different subcarriers location (three, six, nine or 12), depending on the index of the OFDM symbol. All these pilot subcarriers are modulated by a known Pseudo-Random Binary Sequence (PRBS) with a 4/3 boosted signal amplitude compared to data and TPS subcarriers. The organization of the pilot subcarriers is shown in Figure 2. The positioning system described in this paper is based on the processing of the scattered pilot subcarriers (SPS).

In the process of positioning computation, we are interested in the ranges between the receiver and different emitters. These can be obtained by estimating the propagation delay. In DVB-T, the propagation delay is mainly related to the FFT size, *N_FFT_* (which corresponds to the number of subcarriers of the OFDM symbol), the ratio between the Cyclic Prefix length and the useful OFDM symbol length, *ρ_CP_* = *N_CP_*/*N_FFT_*, and the sampling period, *T_samp_*. They are summarized in Table 1 according to the document, [1].

These values determine the maximum delay that a DVB-T receiver can handle. For example, in the 2 K mode with *ρ_CP_* = 1/4 and an 8 MHz channel, the maximum detectable delay is 56 *μ*s, which corresponds to a path difference of about 16.8 km, assuming the propagation velocity is equal to the speed of light in vacuum (*c* = 299,792,458 m/s). Therefore, in general, it is possible to associate to each possible system configuration of Table 1 a maximum allowable path difference, Δ*p_max_*, to avoid ISI.

As shown in Table 1, in the 2 K model with an 8 MHz bandwidth, the sampling interval is 7/64 μs. Multiplying by the speed of light, we have 32 m. By half, it is 16 m. This accuracy can be improved by some interpolation methods. Furthermore, we may have four or more DVB-T emitters in a 2D positioning; the obtained position resolution is acceptable for the urban scenario.

## 3. Positioning Based on DVB-T

It is well known that the position of an object capable of measuring the distances between itself and some reference points can be performed by using the trilateration method; this is the technique typically employed in GNSS [13], and the same method can be utilized when the references points are the emitters of DVB-T signals. To implement the method, the PD has to:

1. identify a reference frame,
2. identify the emitter locations, p*_i_* = (*x_i_*, *y_i_*, *z_i_*), where *i* = 1, …, *N_e_* and *N_e_* are the number of visible DVB-T emitters,
3. measure the ranges, *r_i_*, between each emitter and the PD
4. write and solve the navigation equations.

The implementation of the first and fourth tasks does not present any difference with respect to a classical GNSS receiver; so, it is not described in this paper. The second task is the most challenging, since the signals transmitted by the DVB-T emitters do not contain the station identifier; so, the receiver is not able to associate each received signal to a specific station. This is not an issue for TV reception, but it is a problem for positioning. Notice that the emitter identifier is foreseen by the DVB-T standard, but its transmission is optional; so, it is ignored in this study. The method proposed in this paper to solve this problem is described in Section 3.1. The third task requires the processing of an OFDM signal to estimate the range; so this has to be re-designed with respect to a GNSS receiver.

The next sections will be devoted to the methods used to implement tasks 2 and 3, while the position computation can be implemented by solving the navigation equations with a classical extended Kalman filter (EKF) technique [14,15]. The experimental results shown in this paper have been obtained by using this approach.

### 3.1. Initialization Phase for DVB Ranging

We assume here that in the future, a PD is likely to be equipped with a GNSS sensor as a primary tool and with other sensors able to exploit the nearby signals-of-opportunity. This means that the architecture of a PD will include a hybrid receiver, which computes its position with GNSS and resorts to SoSs only when the GNSS satellites are not visible. Moreover, in the case of DVB-T towers, it is realistic that the PD has a map of their locations (they are indeed available and easy to find also on the web).

**Figure 3.**
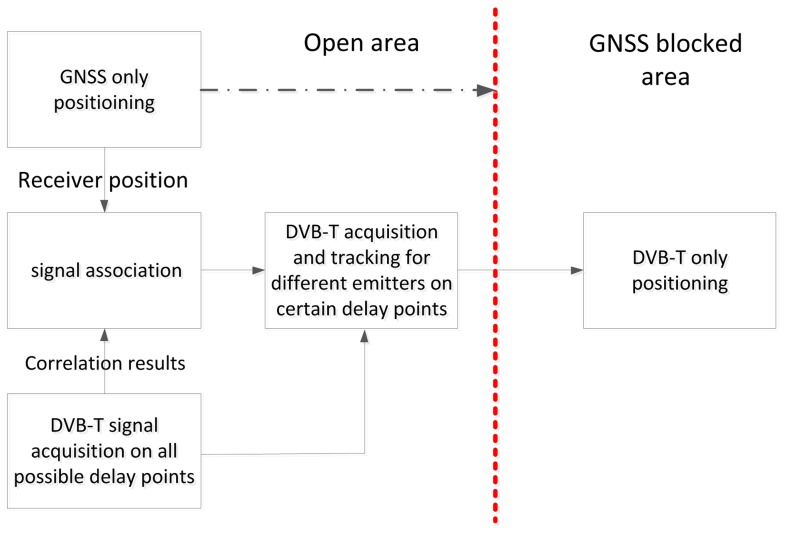
Initialization pahse.

In this scenario, we can suppose that the trilateration with DVB-T-based measurements is generally preceded by a phase of position computation based on GNSS, performed when the PD is in open sky with complete visibility of GNSS satellites. This phase is the standard mode of operation and, at the same time, represents the initialization phase for the position computation based on DVB-T. During this phase, the PD continuously evaluates the ranges, *r_i_*, between each emitter and the PD, by using its own position, provided by GNSS, and the location of the nearby DVB-T emitters, whose map is stored in the PD memory. Therefore, in this phase, the PD can also associate the estimated ranges with the emitter locations, solving the problem of emitter identification, as shown in Figure 3.

Each range can be written at each discrete-time instant, *n*, as:

(3) $$r_{i,G}[n]=N_{i,G}[n]r_{s}+\delta_{i,G}[n]$$

where *N*_*i*,*G*_[*n*] is an integer, *r_s_* is the distance traveled by the DVB-T signal during an OFDM symbol time, *δ*_*i*,*G*_[*n*] is a fractional range with respect to *r_s_* and the subscript, *G*, stands for GNSS. Notice that *N*_*i*,*G*_[*n*] could be also zero, depending on the coverage area of the DVB-T towers. At the same time, the PD can estimate the ranges from the DVB-T emitters by using the SoOs. This can be obtained by measuring the fractional range, *δ*_*i*,*G*_[*n*], as shown in Section 3.2, and by evaluating *N*_*i*,*G*_[*n*] from Equation (3), as:

(4) $$N_{i,G}[n]=\lfloor \frac{r_{i,G}[n]}{r_{s}}\rfloor$$

where ⌊·⌋as the argument of the peak of the correlation function stands for the integer part. Therefore, the PD knows at each instant, *n*, both *r_i_*_,*G*_[*n*] and the range provided by the DVB-T SoOs, which can be written as:

(5) $$r_{i,D}[n]=N_{i,G}[n]r_{s}+\delta_{i,D}[n]$$

and can keep these values aligned. At this point, if the PD enters a GNSS-blocked area at the time instant, *n*_0_, it can start the position computation in DVB-mode by using the range measured at the time instant, *n*_0_ – 1, that is, the range measured during the initialization phase. This represents a hot start for the PD, similar to the one encountered in a PD only working in GNSS-mode.

**Figure 4.**
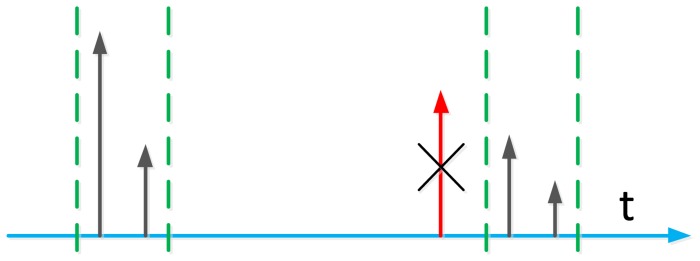
Associating the signals to corresponding receivers.

Another benefit of the initialization phase is that the out bound peaks can be discarded, as shown in Figure 4. The red peak is a multipath of emitter 1, close to the peaks of emitter 2 and stronger than them. However, since it is out of the bound defined by the receiver position, this peak can be recognized as a multipath and, then, discarded.

### 3.2. Range Measurement

In order to obtain a position fix, several ranges are needed in our system. In the case of additive Gaussian noise (AWGN), they can be obtained by the Maximum Likelihood (ML) estimation through correlation. Since the pilot subcarriers are modulated by PRBS, the incoming signals can be correlated with the local generated replicas, similar to the mechanism used by GNSS receivers [16]. However, some differences between GNSS and DVB-T positioning have to be taken into account in the procedure of range estimation, due to the different signal structures.

**Figure 5.**
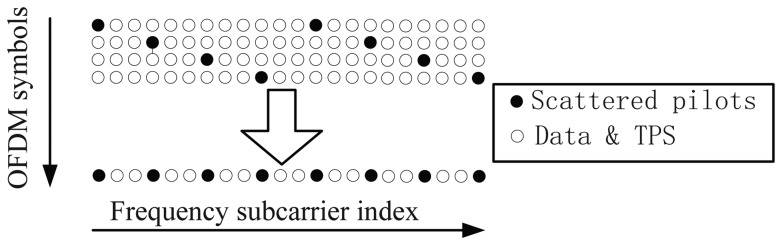
Scattered pilot location of a quadruplet resulting from combining four consecutive symbols.

First of all, the PD has to create a local replica of the SPSs, but the locations of the SPSs of two successive OFDM symbols are different, as shown in Figure 2. Therefore, it is necessary to identify the SPS location of the current symbol, before creating the local replica. A possible identification method is the one proposed in [17] and summarized in Appendix A. This phase can be skipped by adding four successive OFDM symbols together [2], as shown in Figure 5. In this case, the correlation can be performed every four OFDM symbols, and the local replica never changes. This can simplify the receiver structure and speed up the range measurement in the coarse delay estimate. However, after the coarse delay estimate, the correlation should be performed on each OFDM symbol in order to obtain a precise delay estimate.

#### 3.2.1. ML Delay Estimate

It is well known that the ML estimation of the delay of a noisy signal, *s_r_*(*t*) = *s*(*t* − *τ*) + *w*(*t*) (where *w*(*t*) is a realization of an AWGN process), is obtained by correlating *s_r_*(*t*) with a signal, *s*(*t*), generated at the receiver side and by estimating *τ* as the argument of the peak of the correlation function [18]. In our case, this rule has to be applied to the signal at the output of the receiver chain shown in Figure 1.

In the ideal case of a noise-free channel, each received SPS, *p*, of the *k*-th OFDM symbol generates, after FFT demodulation, a value:

(6) $$d_{p}^{k}=c_{p}^{k}\alpha e^{-j2\pi \frac{\mathrm{pn}}{N_{\mathrm{FFT}}}}$$

where *α* is the attenuation introduced by the channel, and it is a real number; and *n* is the unknown delay to be estimated, normalized with respect to *T_samp_*. In the presence of noise, the received value will be:

(7) $${d^{\prime }}_{p}^{k}=d_{p}^{k}+w_{k}$$

where *w_k_* is the noise contribution.

To perform the correlation, a local replica of $d_{p}^{k}$, with a variable delay, *ñ*:

(8) $$P_{p}^{k}=c_{p}^{k}e^{-j2\pi \frac{p\tilde{n}}{N_{\mathrm{FFT}}}}$$

has to be generated and correlated with ${d^{\prime }}_{p}^{k}$. This correlation can be performed in the frequency domain, as described in [2,6], as:

(9) $$R_{M}(m)=\frac{1}{N_{p}}\sum_{p\in p_{s}(k)}{d^{\prime }}_{p}^{k}P_{p}^{k*}$$

where *N_p_* is the number of SPSs in the OFDM symbol, (·)* denotes a complex conjugate, *m* = *n* − *ñ* is the delay offset between the received signal and the local replica and *p_s_*(*k*) is the index set of the SPSs of the *k*-th OFDM symbol.

#### 3.2.2. Correlation in the Ideal Case

In the ideal case of a single emitter in a noise-free channel, without multipath and frequency offset, the correlation, *R_M_*(*m*), can be analytically evaluated for *m* ∈ Δ*_m_*, where Δ*_m_* is a discrete-time interval containing all the possible delays between the incoming signal and the local replica (expressed in number of samples). By substituting ${d^{\prime }}_{p}^{k}=d_{p}^{k}$ in Equation (9), it is possible to show [9] that the absolute value of *R_M_*(*m*) is:

(10) $$\left|R_{M}(m)|=\frac{16}{9}\sigma_{p}^{2}\alpha |\frac{\sin(\pi \mathrm{Bm})}{\sin(\frac{\pi \mathrm{Bm}}{N_{p}})}\right|$$

where $\sigma_{p}^{2}$ is the power of the data and TPS subcarriers and *B* is defined as:

(11) $$B=\frac{P_{I}N_{p}}{N_{\mathrm{FFT}}}$$

where *P_I_* is the interval between two adjacent SPSs. Notice that *R_M_*(*m*) is a periodic function with a period equal to *N_FFT_*/*P_I_*. This period restricts the range of delays that can be estimated. From this point of view, the quadruplets of Figure 5 are more convenient as *P_I_* decreases from 12 to three.

In order to mitigate the side lobe of the correlation function, we use a windowing technique. By adopting a hamming window, the correlation becomes:

(12) $$R(m)=\frac{1}{N_{p}}\sum_{p\in p_{s}(k)}{d^{\prime }}_{p}^{k}P_{p}^{k*}w(p)$$

where *w*(*p*) = 0.53836 − 0.46164 cos(2*πp*/(*K* − 1)) is the hamming window and *K* is the number of non-null subcarriers.

#### 3.2.3. Delay Estimate in the Case of Multiple Emitters

In a scenario with more than one emitter, the correlation is characterized by the presence of several peaks, each one corresponding to a delay, *m_l_* (with *l* = 1, ⋯, *N_e_*), introduced in the correlation by each specific emitter. Moreover, each emitter can generate both the line of sight (the only path of interest for positioning) and other reflected signals. The peak positions can be evaluated by adopting a threshold mechanism applied in the regions of expected delays. The threshold can be calculated as stated in the Neyman-Pearson theorem. However, in our simulation, since we are interested in the performance of the Position, Velocity and Time (PVT) computation and we know that the signals are present, we skip the phase of signal detection, and we simply estimate the propagation delay by considering the position of the peak.

In our experiments, we have used a mechanism consisting of two stages; the first one, called *acquisition*, provides a coarse delay estimate, which will be refined by a tracking stage in the second stage. The method adopted in the acquisition stage is based on the comparison of the correlation obtained from the measured samples, with the ideal expected correlation, which is, in the ideal case, of no noise, and other interfering signals, of the type:

(13) $$R_{N_{e}}(m)=\overset{N_{e}}{\underset{l=1}{\text{∑}}}R(m-m_{l})$$

The criterion adopted in the acquisition stage is the minimization of the mean squared error between the measured correlation, *R_M_*(*m*), and the ideal correlation, expressed as:

(14) $$\hat{m}=\arg\min_{\bar{m}}\overset{K}{\underset{m=1}{\text{∑}}}{\left\|R_{M}(m)-\overset{N_{e}}{\underset{l=1}{\text{∑}}}R(m-{\bar{m}}_{l})\right\|}^{2}$$

where ║ · ║^2^ denotes the two-norm of a vector, *K* is the number of correlation points, m̄ is a test vector, **m̄** = {*m̄*_1_*m̄*_2_ ⋯ *m̄_Ne_*}, whose elements are variable delays, *m̄_l_*, introduced for performing the minimization procedure indicated in Equation (14), and **m̂** is the vector of the estimated delays.

The minimization can be done by adopting the Matching Pursuit (MP) algorithm [19], which estimates the delays by searching for the correlation peaks in an iterative way The search space should be the whole range, Δ*_m_*, but in our application, the size of the search interval, Δ*_m_*, can be greatly reduced, thanks to the initialization phase, which allows a prediction of the peak locations. In this way, the estimation of the delays is done only in restricted intervals around the true delays, *m_l_*. This allows a hot start of the DVB mode, which presents another important advantage: the majority of peaks due to multipaths are discarded, as they fall outside the search space identified during the initialization phase.

Once the coarse delay estimation has been achieved by the acquisition stage, the tracking stage can be used to refine this estimation by using an early-late delay lock loop (DLL) similar to the one adopted in a GNSS receiver. An example of DLL design can be found in [6,9]. One difference between the tracking used here and that in GNSS is the re-acquisition phase. This function can be used to refine the tracking performance as the correlation peak jumps from one delay point to the adjacent point. This phase is described in Section 3.2.4.

#### 3.2.4. Re-Acquisition Phase

**Figure 6.**
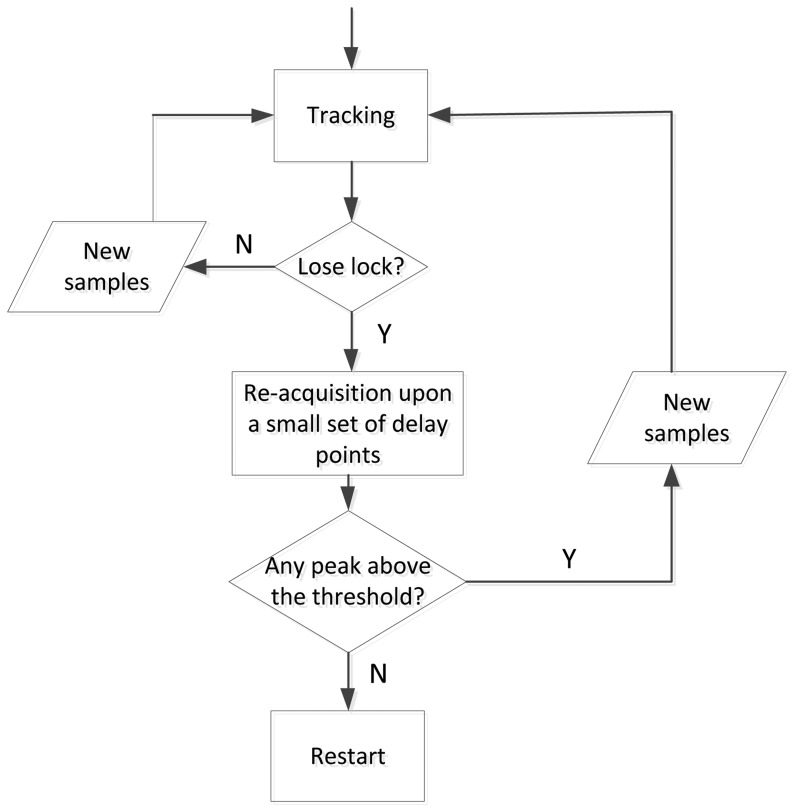
The flow diagram of the re-acquisition stage.

Since there is only one sample for each subcarrier, the output of the discriminator will change rapidly once the correlation peak jumps from one delay point to the adjacent one. This is different from the tracking process used in a GPS receiver, where the receiver can track the signal smoothly from one chip to the next one. In order to solve this problem, a re-acquisition function is implemented. If the output of the discriminator is above a given threshold, the receiver will enter the re-acquisition stage. This stage is similar to the acquisition stage, but the correlation is only calculated upon a small set of delay points, which are centered on the delay obtained from the tracking process. Here, we choose three delay points. In fact, the receiver trajectory is continuous, and then, the correlation peak can only jump from one delay point to one of the adjacent two points (depending on the increase or decrease of the range). If all the correlation results are below a given threshold, the tracking is considered as unlocked. The receiver will restart from the first step. Otherwise, the receiver goes back to the tracking process with the new estimated delay obtained in the re-acquisition stage, as shown in Figure 6.

#### 3.2.5. Final Considerations

It is well known that the minimum number of reference points for performing the trilateration process in a 3-D space is four. In practice, in most applications, three reference points could be enough, as the intersection of three spheres gives two solutions, one of which is not realistic and can be discarded. Moreover, some aiding data can be provided during the initialization phase, such as the synchronism, and the user altitude, further reducing the minimum number of DVB-T emitters.

In a more realistic situation, three or two DVB-T signals should be sufficient, considering that in most applications, some data can be considered quite constant (*i.e.*, altitude) during short/medium GNSS outages, especially in a vehicular scenario. Moreover, if the hybrid GNSS/DVB receiver can track its position in an area where GNSS satellites randomly appear and disappear, the position provided by DVB-T can be used to allow a very hot start of the GNSS-based positioning, even if the GNSS satellites are visible in a very short time interval. In general, we can say that a hybrid GNSS/DVB receiver could be used for both a hybrid trilateration and for mutual assistance of the two separate positioning systems. This paper is devoted to this second aspect, and the simulation experiments with four emitters have been done to prove the feasibility of a positioning method based on synchronous OFDM DVB-T signals, while the role of GNSS is only the initialization of the DVB-T-based position computation.

## 4. Path Ambiguity Solution

**Figure 7.**
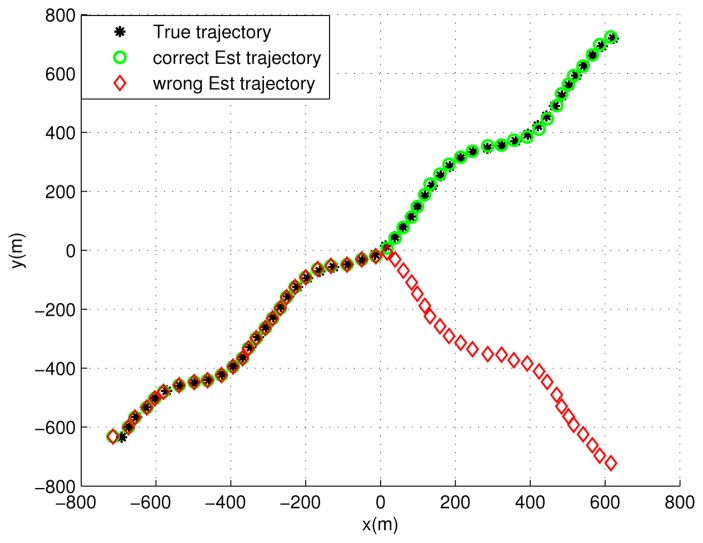
True and estimated trajectories.

In this section, two methods are presented to solve the path ambiguity problem, which is caused by wrong signal association during the user's motion, which can be observed from Figure 7. One is based on pseudorange (PR) comparison and the other on a Doppler aiding decision. These two methods benefit from a single visible GNSS satellite. The pseudorange comparison method compares the pseudorange estimated from the GNSS signal and the corresponding value computed by using the known satellite position and the receiver position in the alternative paths. The Doppler aiding decision exploits the Doppler effect in the GNSS signal to assist the receiver velocity and trajectory decision.

### 4.1. Pseudorange Comparison

When a user receives two or more DVB-T signals with similar or exactly the same propagation delays, it can exploit the visibility of a single GNSS satellite to solve the path ambiguity problem by comparing the ranges. This method is described by considering the case of two DVB-T signals with the same propagation delay Therefore, two possible positions are calculated.

**Figure 8.**
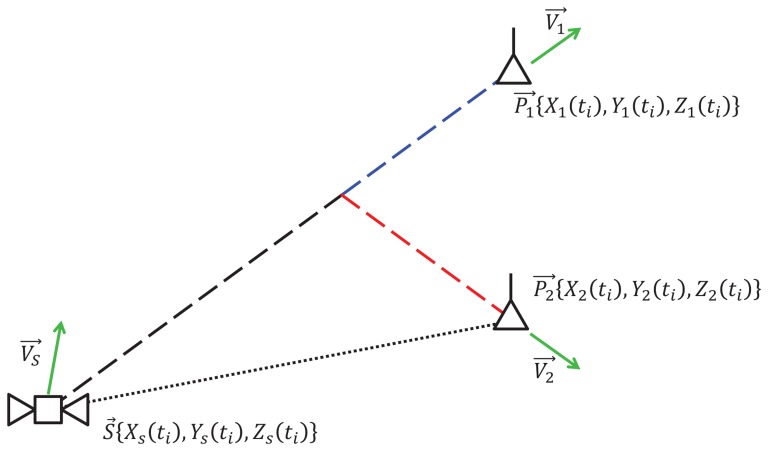
Illustration for pseudorange comparison.

In Figure 8, we suppose that the GNSS satellite is at position, *S⃗*{*X_s_*(*t_i_*),*Y_s_*(*t_i_*), *Z_s_*(*t_i_*)}, with velocity, $\vec{\upsilon_{s}}$, at time *t_i_*. The receiver is at position, *P⃗*_1_{*X*_1_(*t_i_*), *Y*_1_(*t_i_*), *Z*_1_(*t_i_*)}, with velocity, $\vec{\upsilon_{1}}$. Besides this, the receiver may also produce a wrong position estimate, which is *P⃗_2_*{*X_2_*(*t_i_*), *Y_2_*(*t_i_*), *Z_2_*(*t_i_*)} with velocity, $\vec{\upsilon_{2}}$. Therefore, at each time, *t_i_*, the receiver can calculate the distance between itself and the observed satellite. Taking into account the two estimated positions, the two ranges, *d*_1_ and *d_2_*, are evaluated as:

(15) $$d_{1}=\sqrt{{\left(X_{s}(t_{i})-X_{1}(t_{i})\right)}^{2}+{\left(Y_{s}(t_{i})-Y_{1}(t_{i})\right)}^{2}+{\left(Z_{s}(t_{i})-Z_{1}(t_{i})\right)}^{2}}$$

and:

(16) $$d_{2}=\sqrt{{\left(X_{s}(t_{i})-X_{2}(t_{i})\right)}^{2}+{\left(Y_{s}(t_{i})-Y_{2}(t_{i})\right)}^{2}+{\left(Z_{s}(t_{i})-Z_{2}(t_{i})\right)}^{2}}$$

When the pseudorange comparison method is active, the user can compare *d*_1_ and *d*_2_ with *ρ*′ = *ρ* + *n*, which is the range derived from the GNSS signal, while *n* is the error source, which is modeled with a Gaussian distribution. It is worth mentioning that the local clock error can be estimated when the user is in open sky. Therefore, the range can be obtained instead of the pseudorange. The distance differences can be computed as:

(17) $$\Delta d_{1}=d_{1}-\rho^{\prime }$$

(18) $$\Delta d_{2}=d_{2}-\rho^{\prime }$$

If one of them is above a given threshold, the corresponding position can be marked as fake, and this path will be discarded in the further calculation. We choose three times the standard deviation of the satellite range error as a reasonable threshold, considering that the distance difference corresponding to the correct path will be less than the threshold, with a probability of 99%.

### 4.2. Doppler Aiding Decision

**Figure 9.**
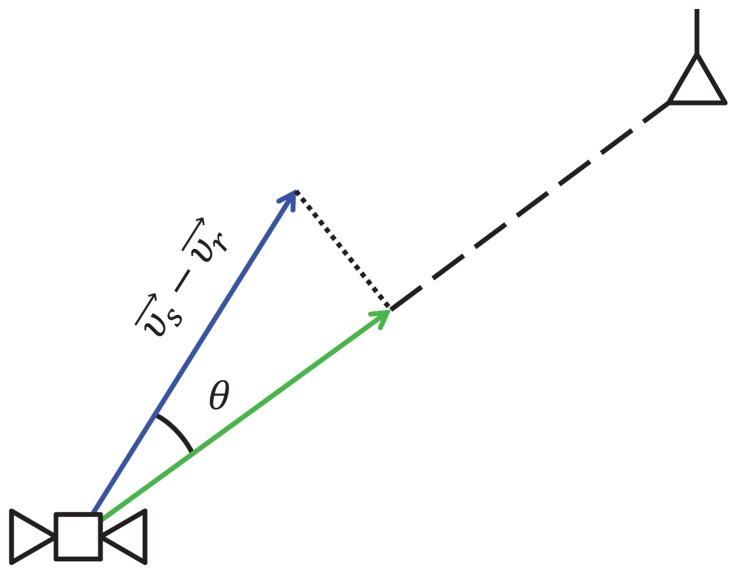
Doppler effect.

Another method to select the correct path is based on the Doppler effect, due to the relative motion between the satellite and the receiver. The Doppler effect is generally expressed in terms of frequency shift, on the basis of the quantities shown in Figure 9. If $\vec{\upsilon_{r}}$ is the true velocity of the receiver, then the frequency shift can be written as:

(19) $$\Delta f=\frac{\|\vec{\upsilon_{s}}-\vec{\upsilon_{r}}\|\cos\theta }{\lambda }$$

where $\left\|\vec{\upsilon_{s}}-\vec{\upsilon_{r}}\right\|$ is the magnitude of the relative velocity of the receiver with respect to the satellite, *θ* is the projection angle of the satellite-receiver relative velocity vector to the Line of Sight (LOS) vector nd *λ* is the nominal wave length of the GNSS signal.

During the path ambiguity phase, the receiver can compute a vector containing the coordinates of position and velocity for each path: one of them is correct, and the others are fake. For each possible vector, the receiver can then calculate the corresponding Doppler effect expressed as:

(20) $$\Delta f_{r}=\frac{\|\vec{\upsilon_{s}}-\vec{\upsilon_{r}}\|\cos\theta_{r}}{\lambda }$$

where *υ⃗_r∈_*_{1,2}_ is the possible velocity for the two positions and *θ_r∈_*_{1,2}_ is the projection angle of the satellite-receiver relative velocity vector to the LOS vector for the two possible positions, which can be obtained as:

(21) $$\theta_{r}=\arccos\frac{\left(\vec{\upsilon_{s}}-\vec{\upsilon_{r}})\cdot ({\vec{P}}_{s}-{\vec{P}}_{r}\right)}{\left\|\vec{\upsilon_{s}}-\vec{\upsilon_{r}}\right\|\cdot \left\|{\vec{P}}_{s}-{\vec{P}}_{r}\right\|}$$

It is known that a GNSS receiver is able to estimate a Doppler shift, Δ*f_GNSS_*, from the received signal. Therefore, by comparing the values of Δ*f_r_* with Δ*f*_GNSS_, the true trajectory can be selected. In this method, we do not need to consider the DVB-T receiver clock offset to calculate Δ*f_r_*, since it is directly related to the estimated position.

## 5. Simulation Results

In our application, we assume that the PD is able to receive the signals broadcast by different DVB-T emitters belonging to the same SFN. To analyze how the PD can handle these signals, we assume a scenario with *N_e_* emitters. The distance between the PD and the *i*-th emitter is denoted as *d_e_*_,*i*_, the distance, *d_e_*_,1_, is associated to the closest emitter and the relative distance, Δ*_i_* = *d_e_*_,1_ − *d_e_*_,*j*_, with *j* = 2, ⋯, *N_e_*, is introduced. If the PD is able to receive the closest signal (used as the reference signal), the signals broadcast by emitters with Δ*_i_* < Δ*p_max_* can be detected, and the relative delays of these emitters can be estimated by using the same techniques used for the reception of the useful signal. The emitters with Δ*_i_* > Δ*p_max_* are seen as noise. In theory, in positioning applications, also, these distant emitters could be used, trying to detect them with a dedicated processing. However, they are generally blinded by the close emitters; so, in our study, they have not been considered. In our method, we assume that the PD starts working in DVB-mode after an initialization phase, which allows the PD to evaluate *N_e_* and the initial distances, *d*_*e*,*i*_.

One important aspect for positioning is the Doppler effect. Since all the emitters are fixed, the Doppler effect is introduced by the receiver's motion. In [20], this effect on the DVB-T system has been investigated. The results show that for 2 K QPSK, more than 400 km/h is tolerable, but for 8 k 64 QAM, the tolerance is less than 50 km/h. Therefore, in our application, especially in the 2 K mode experiment, the Doppler effect is negligible.

### 5.1. Simulation Scenario

**Figure 10.**
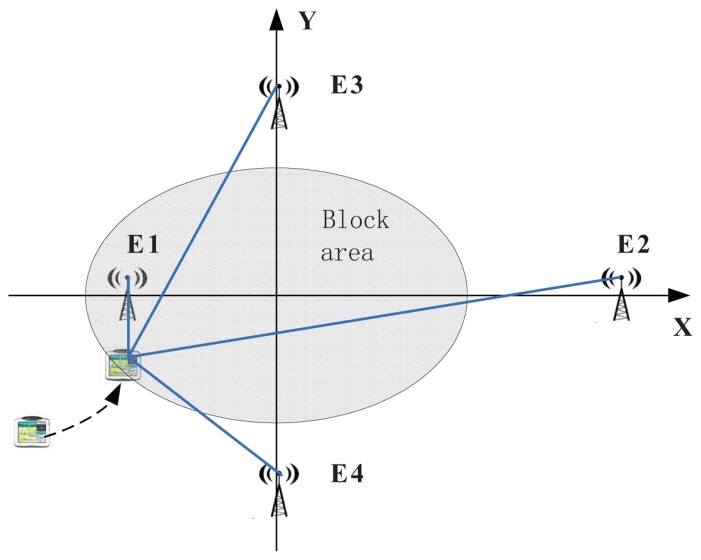
Analyzed scenario.

The simulation experiments have been done for the scenario shown in Figure 10, where the user can receive four DVB-T signals from four emitters that belong to an SFN, and no GNSS satellites are visible when the user enters the grey area indicated in the figure. The user moves along a path with an initial location outside the grey area. In our simulation, four DVB-T emitters are placed.

In the scenario of Figure 10, four DVB-T emitters of the same SFN are placed on the four semi-axes. They transmit the same signals on the same frequency, with the parameters summarized in Table 2.

For the signal generation, we have used the Anritsu MX3700 generator, which is a laboratory Radio Frequency (RF) generator that can be modulated by an array of complex baseband samples. As an option, a DVB-T/H sample generation software is sold. We used such software to generate DVB-T-compliant baseband signals used as simulation sources. The receiver has a constant speed equal to 100 m/s, while the direction of the velocity changes from time-to-time. The signal received along the path has been simulated by combining shifted and attenuated versions of the generated signal. The delays have been simulated by shifting the FFT window according to the corresponding ranges between the user and the emitters. The SNR is simulated according to Equation (22):

(22) $$C/N_{0}=\frac{E[{\left|s(t)\right|}^{2}]}{N_{0}L_{u}}$$

where *s*(*t*) is the transmitted signal; *N*_0_ is the noise floor; *L_u_* is the signal attenuation according to the Hata model for urban areas, which is:

(23) $$L_{u}=69.55+26.16\log(f)-13.82\log(h_{B})-C_{H}+[44.9-6.55\log(h_{B})]\log(d)$$

where *C_H_* depends on the city size. For small and medium sized cities, it is:

$$C_{H}=0.8+(1.1\log(f)-0.7)h_{M}-1.56\log(f)$$

while, for large cities, *C_H_* is:

$$C_{H}=\{\begin{array}{ll} 8.29{\left(\log(1.54h_{M})\right)}^{2}-1.1, & \mathrm{if}\;150\leq f\leq 200\\ 3.2(\log{\left(11.75(h_{M})\right)}^{2}-4.97, & \mathrm{if}\;200\leq f\leq 1500 \end{array}$$

where *L_u_* is the path loss in urban areas in dB, *h_B_* is the height of emitters in meters, *h_M_* is the height of the receiver antenna in meters, *f* is the frequency of transmission in MHz, *C_H_* is the antenna height correction factor and *d* is the distance between the base station and receiver in kilometers.

Here, we suppose all the DVB-T transmitters are placed on the same height at 100 m. Furthermore, since all the emitters use the same frequency, the propagation loss changes only with the distance. The SNR of the received signal has been set equal to 10 dB, where the distance between the user and the emitter is equal to 5 km. The SNR in the other points is calculated according to Equation (23).

The position is computed in two dimensions, regardless of the altitude, every 0.2 s.

### 5.2. First Experiment

In the first experiment, the position is evaluated in the absence of multipath and by ignoring the propagation loss. The idea is to test only the capability of a system based on DVB-T, and GNSS is only used in the initialization phase. The simulation length is set to 20 s. The four DVB-T emitters are placed in this way: the two emitters (E1 (−1,000,0) and E2 (8,000,0)) placed on the x-axis have quite different distances with respect to the origin of the reference system, while the other two (E3 (0,4,000) and E4 (0,−4,000)) on the y-axis are symmetric with respect to the origin.

**Figure 11.**
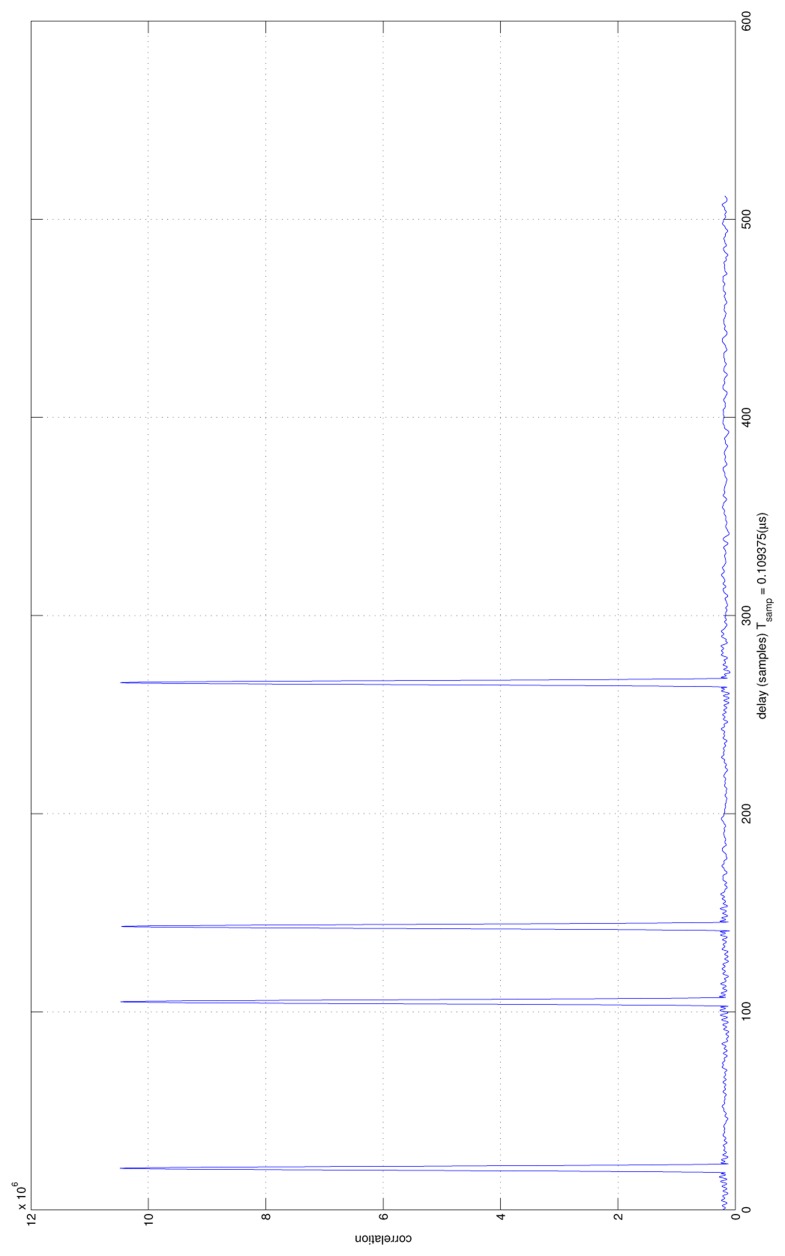
Acquisition results (correlation).

Figure 11 shows the acquisition results. Four correlation peaks are visible. They correspond to four different propagation times with respect to the four DVB- T emitters at the initial location, from where the user enters the obstructed area. Since the propagation loss has not been considered, the four correlation peaks have the same amplitude.

Figure 7 presents the estimated trajectory. In order to make the figure more visible, the trajectory plots are done every 0.4 s, which means that one estimated point between two presented points is dropped. In the figure, two different estimated trajectories are visible. The green circles always stay close to the true trajectory, while the red diamonds deviate from the true location after the user reaches the origin of the reference system. This is because when the receiver reaches the origin, it receives four signals, and two of them have the same propagation time (E3 and E4 are symmetric with respect to the origin). Then, two different situations may exist in the absence of additional information:

1. The receiver still makes the correct signal association after this point, and the receiver will estimate the trajectory correctly. This is similar to the case of signals from different emitters with significantly different propagation times. In this case, the simulation results show good performance, even in highly dynamic scenarios (e.g., 100 m/s), as shown by the green circles. In our simulation, the receiver achieves a performance with a Root Mean Square (RMS) = 6.8 m, Mean error = 6.0747 m and standard deviation *σ_p_* = 3.0559 m.
2. The receiver makes a wrong signal association after reaching the origin of the reference frame, which means the signal emitted by E3 is associated with E4, and the signal of E4 is considered as coming from E3. In this case, the estimated trajectory deviates from the true one, as shown by the red diamonds.

Since we cannot know the true trajectory in the real cases, some additional information (e.g., the cell ID information, map information for map matching, signals from other system) is needed to determine which trajectory is correct in the case that two or more signals have a similar propagation time. Two methods have been introduced in Section 4.

### 5.3. Experiment Considering the Hata Model for Urban Areas

In this experiment, the Hata model introduced in Section 5 is used. Additionally, the path ambiguity problem has been solved by the methods introduced in Section 4. In order to simulate a large range of SNR, the simulation length is set to 100 s, and the two emitters on the y-axis are located so as to have different distances with respect to the origin.

**Figure 12.**
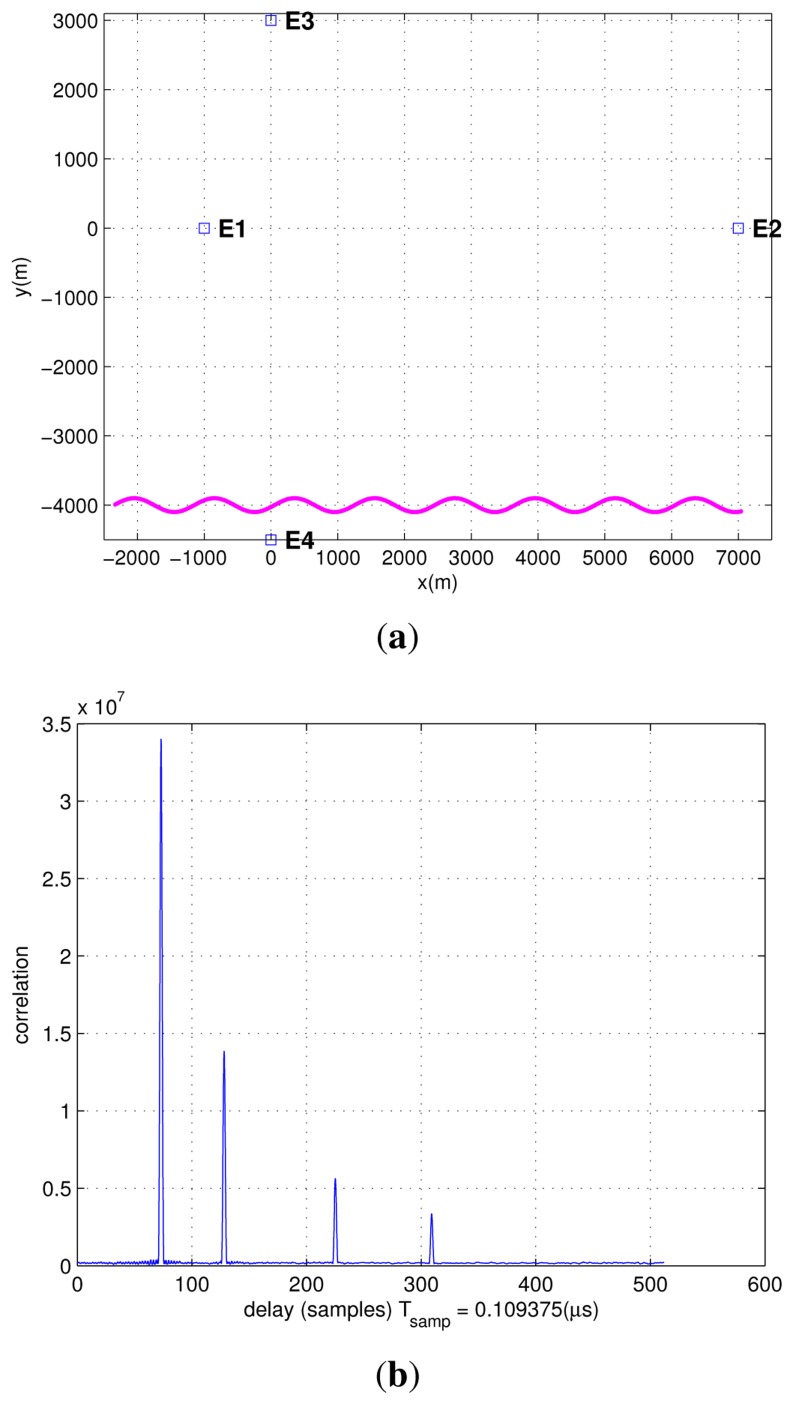
Correlation results in the Hata model.

Figure 12(a) presents the trajectory and the positions of the four emitters, and Figure 12(b) presents the correlation results at the initial point of the trajectory in the Hata model. The four correlation peaks have different amplitudes, depending on the ranges between the emitters and the PD. From this figure, we can see that the amplitude of the correlation peak decreases as the distance between the PD and the emitter increases. Therefore, when the distance increases too much, even if it is less than Δ*p_max_* (the maximum detectable range for one DVB-T signal), the ranging operations could become critical if the SNR becomes too low.

**Figure 13.**
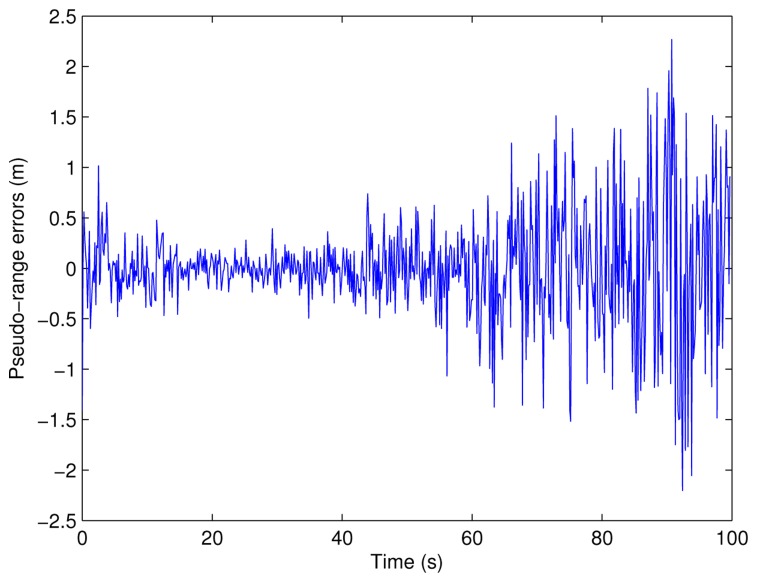
Pseudo-range error for emitter E4.

By multiplying the estimated delay by the speed of light, we obtain the range:

(24) $$r_{m}[n]=r_{t}[n]+\epsilon_{r}[n]$$

where *n* is the discrete time normalized by the OFDM symbol duration, *r_m_*[*n*] is the pseudo-range measurement, *r_t_*[*n*] is the true pseudo-range and *ϵ_r_*[*n*] is the range error due to noise. The range error for emitter E4 is shown in Figure 13, from which it can be easily observed that the error depends on the SNR, which, in turn, depends on the distance between the emitter and the PD.

In this simulation, the estimated position can be expressed as:

(25) $$\begin{array}{l} P_{x,m}[n]=P_{x}[n]+\epsilon_{x}[n]\\ P_{y,m}[n]=P_{y}[n]+\epsilon_{y}[n] \end{array}$$

where *n* is the estimation time, *P_i_*[*n*], with *i* ∈ {*x*, *y*}, is the true value of the PD position on the i-axis, *P_i_*_,*m*_[*n*] is the corresponding estimated value and *ϵ_i_*[*n*] is the estimation error. The total positioning error can be calculated through the following equation:

(26) $$\epsilon [n]=\sqrt{\epsilon_{x}^{2}[n]+\epsilon_{y}^{2}[n]}$$

**Figure 14.**
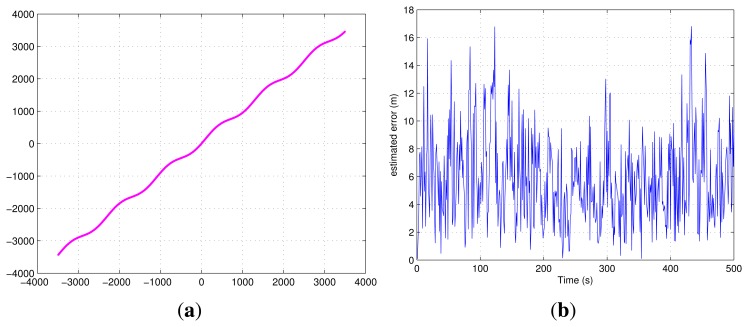

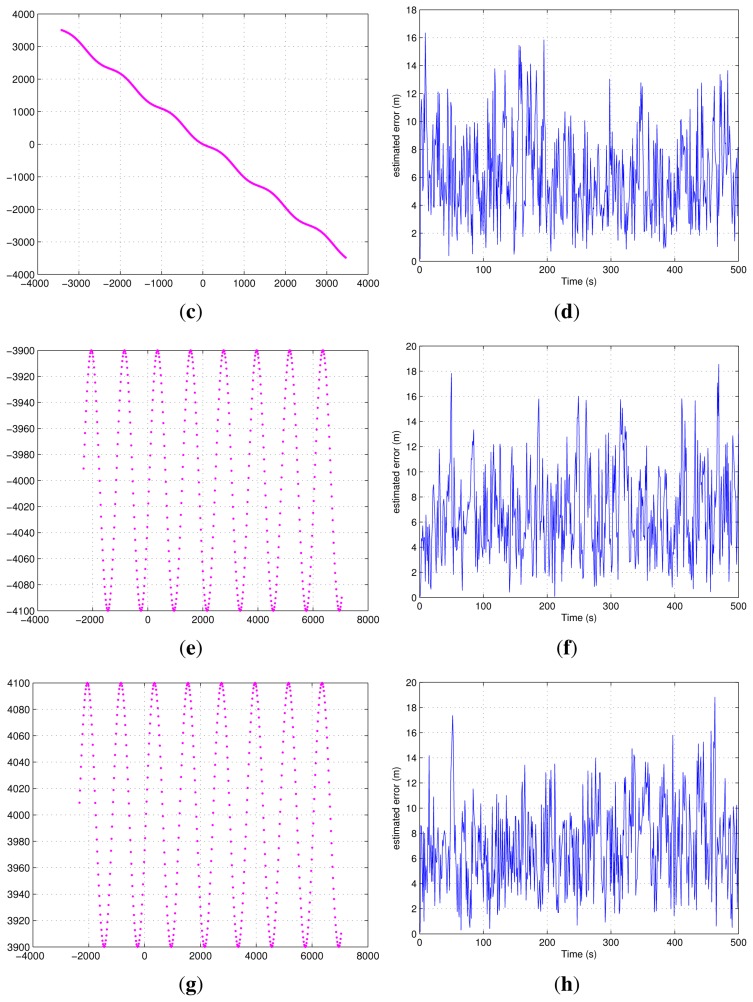
Different trajectories and the positioning errors.

Several different trajectories have been simulated. The true trajectories and the positioning errors, *ϵ* [*n*], are shown in Figure 14, from which no significant dependence on the SNR can be observed. The reason is that along the path, the power of some received signals decreases, while that of others increases, so giving approximately a stationary performance in terms of positioning error.

### 5.4. Experiment with Multipath

In this section, we simulate the Rayleigh channel for multipath, which is suggested by the DVB-T standard [1] for portable reception. Since the method presented in this paper relies on the LOS, we add the LOS signal in this model. The related power, delay and phase values can be found in [1]. In order to simplify the simulation, we delete the multipath signals, which have a time delay lager than 15 OFDM samples with respect to the LOS signal, corresponding to a 492 m separation in 2 K mode, based on the fact that long delay multipaths can be filtered out on the basis of the initial information.

**Figure 15.**
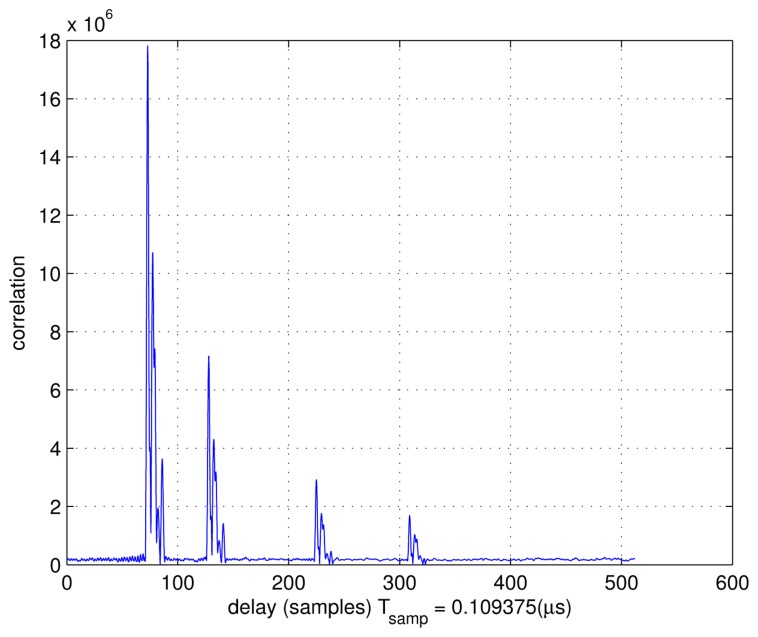
Acquisition results with multipath channel.

**Figure 16.**
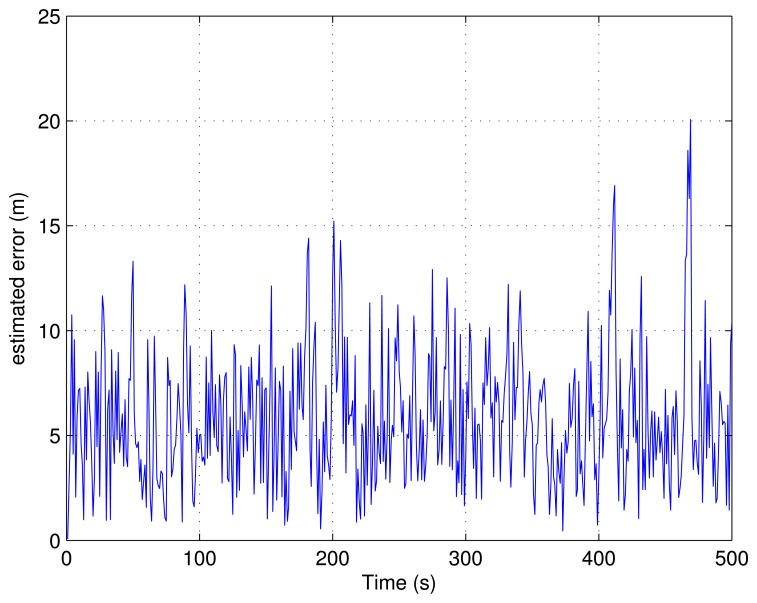
The position estimation errors with multipath channel.

The correlation with the Rayleigh multipath channel is shown in Figure 15. The Hata propagation loss model is also introduced. The trajectory estimation error is shown in Figure 16. From this error plot, we see that the position error is comparable with the errors in the absence of multipath. This is probably due to the fact that the LOS is present with the strongest signal power. There are four emitters visible, which can also improve the performance in difficult environments for 2-D positioning.

### 5.5. Experiments with Different SNR

**Figure 17.**
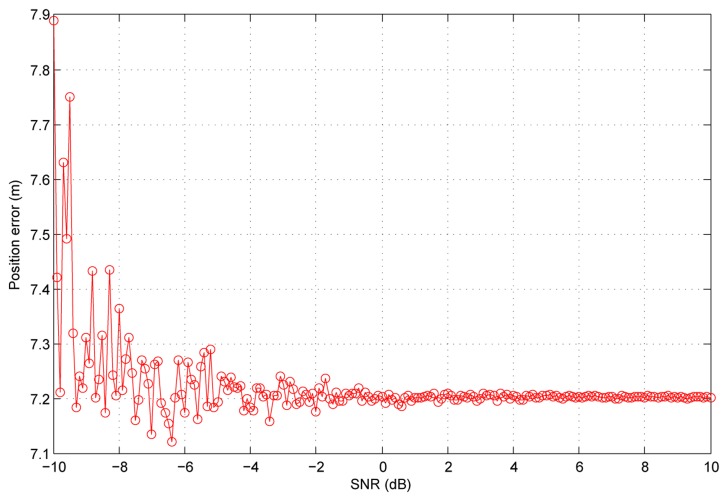
The position errors in different SNR.

In this section, we simulate a scenario to test the performance in different SNR. In this scenario, all the received signals have the same SNR, which changes from −10 dB to 10 dB for different simulations. Additionally, the position errors are shown in Figure 17. In this figure, we can see that when the SNR is low, the position errors are slightly worse and the variance is big. When the SNR goes higher, the position errors converge to a constant. One thing worth noticing is that the DVB-T signals power is very high, above 10 dB, normally due to the error probability requirement of TV service. Additionally, it is normal for the DVB-T receiver to receive signals with an SNR higher than 10 dB.

### 5.6. Experiments with One Transmitter Unsynchronized

In this part, we simulate the scenario in which there is one transmitter unsynchronized. We suppose emitter 1 is unsynchronized with the other three emitters, which are synchronized with GPS time during the position calculation. Different time offsets are tested; while in each simulation, the time offset stays constant. The four trajectories used in Section 5.3 are also simulated here.

**Figure 18.**
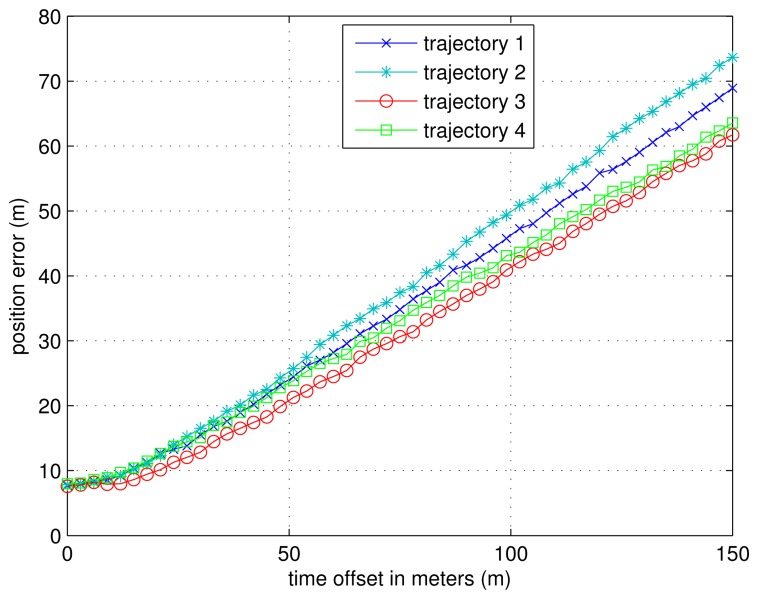
The position estimation errors with one transmitter unsynchronized.

In Figure 18, the mean errors with respect to different time offsets are plotted for the four trajectories. We can see that the position error increases, with the time offset increasing for all four trajectories.

## 6. Conclusions and Future Work

In this paper, a positioning method based on DVB-T Single Frequency Networks has been presented. This method uses the scattered pilot subcarriers of OFDM symbols to measure the Time of Arrival (ToA) of the signals transmitted by the DVB-T emitters. In our study, we suppose that the user is equipped with a hybrid GNSS-DVB-T device. GNSS is used in a first initialization phase to solve the ambiguities referring to the various DVB-T emitters. When this phase is completed, the user position can be obtained by using DVB-T signals when the user enters a GNSS-blocked area. (Note that transmission of the emitter ID would be very useful for positioning. Since it is optional, it has not been considered in the study, but obviously, it would further simplify initialization and tracking of DVB signals.)

The method has been tested by simulation in a dynamic scenario. The simulation results show that a position mean error = 6.0747 m can be achieved if the user can correctly associate the signals to the emitters. If the receiver makes a wrong association, some additional information is needed to determine which trajectory is the correct one. The Hata model has been used to simulate emitters with different SNRs, and a Rayleigh channel has been also introduced to take into account the multipath. The results shows that the range error changes with respect to SNR, while the position estimates do not change significantly along the trajectory. However, if one of the DVB-T emitters is unsynchronized, the position error will increase as the time offset increases.

The result of this study is that the structure of the DVB-T SFN signals is such that a PD working with DVB-T signals can be conceived. At this point, the performance of the positioning algorithms in the presence of different errors sources has to be evaluated. In our future work, a more realistic multipath channel will be considered, and an analysis taking into account other error sources will be performed.

## Figures and Tables

**Table 1.** DVB-T parameters.

**Parameter**	**Possible Values**
*N_FFT_*	2,048 (Mode 2 K)
	4,096 (Mode 4 K)
	8,192(Mode 8 K)
*ρ_CP_*	1/32, 1/16, 1/8, 1/4
*T_samp_*	7/64 μs (8 MHz))
	1/8 μs (7 MHz)
	7/48 μs (6 MHz)
	7/40 μs (5 MHz)

**Table 2.** Parameters of DVB-T signals. FFT, Fast Fourier Transform; CP, Cyclic Prefix; SNR, signal-to-noise ratio.

**Parameters**	**Values**
FFT size *N_FFT_*	2,048
CP length	$\frac{1}{4}(56\;\mu \text{s})$
Signal bandwidth	8 MHz
Length of simulation	20 s, 100 s
Symbol duration *T_symb_*	280 μs
Sampling period *T_samp_*	109 ns
SNR	10 dB

## Appendix A. Scattered Pilot Subcarrier Detection

The detection algorithm proposed in [17] is used to identify the location of the current SPSs.

This algorithm makes use of the signal property that an OFDM symbol has the same scattered pilot subcarriers as the OFDM symbol located four symbols earlier. The correlation of these two OFDM symbols is computed, obtaining:

(27) $$S(m)=\sum_{p\in p_{s}(m)}d_{p}^{k}{\left(d_{p}^{k-4}\right)}^{*},m\in \{3,6,9,12\}$$

where *p_s_*(*m*) is the set of the index of the *m*-th pattern of the scattered pilot subcarriers and (·)* denotes the complex conjugate.

The maximum value of the four correlations gives the location of the scattered pilot subcarriers of the *k*-th OFDM symbol, which is:

(28) $$\hat{m}=\arg\max_{\bar{m}}|S(\bar{m})|$$

where *m̂* ∈ {3, 6, 9, 12} denote different scattered subcarrier sets with different first scattered pilot subcarrier locations. For example, *m̂* = 3 indicates that the location of the first scattered pilot subcarrier is three. The scattered pilot subcarriers of the other OFDM symbols can be obtained by using the rule described in [1].
